# TIMM8A is associated with dysfunction of immune cell in BRCA and UCEC for predicting anti-PD-L1 therapy efficacy

**DOI:** 10.1186/s12957-022-02736-6

**Published:** 2022-10-07

**Authors:** Xiaoyu Zhu, Zile Yuan, Sheng Cheng, Hongyi Wang, Yuxuan Liao, Dawei Zhou, Zhiqiang Wu

**Affiliations:** 1 Department of Dermatology, The Fourth Hospital of Changsha, Changsha, Hunan 410000 China; 2 Hunan Key Laboratory of Oral Health Research, Hunan 3D Printing Engineering Research Center of Oral Care, Hunan Clinical Research Center of Oral Major Diseases and Oral Health, Xiangya Stomatological Hospital, and Xiangya School of Stomatology, Central South University, Changsha, Hunan 410008 China; 3 Xiangya School of Medicine, Central South University, Changsha, Hunan 410013 China

**Keywords:** TIMM8A, Breast cancer, Uterine corpus endometrial cancer, Anti-PD-L1

## Abstract

**Background:**

TIMM8A is a protein-coding gene located on the X chromosome. There is evidence that TIMM8A plays an important role in mitochondrial morphology and fission. Studies have shown that mitophagy and fission could affect the function of immune cells. However, there is currently no research on this gene’s role in cancer occurrence and progression.

**Methods:**

TIMM8A expression was analyzed via the Tumor Immune Estimation Resource (TIMER) site and UALCAN database. We evaluated the influence of TIMM8A on clinical prognosis using Kaplan-Meier plotter, the PrognoScan database, and Human Protein Atlas (HPA). The correlations between TIMM8A and cancer immune infiltrates were investigated via TIMER. Tumor Immune Dysfunction and Exclusion (TIDE) was used to evaluate the potential of tumor immune evasion. Functions of TIMM8A mutations and 50 genes significantly associated with TIMM8A mutations in breast cancer (BRCA) and uterine corpus endometrial cancer (UCEC) were analyzed by GO and KEGG in LinkedOmics database.

**Results:**

We investigated the role of TIMM8A in multiple cancers and found that it was significantly associated with poor prognosis in BRCA and UCEC. After analyzing the effect of TIMM8A on immune infiltration, we found Th2 CD4+ T cells might be a common pathway by which TIMM8A contributed to poor prognosis in BRCA and UCEC. Our results suggested that myeloid-derived suppressor cells (MDSC) and tumor-associated M2 macrophages (TAM M2) might be important factors in immune evasion through T cell rejection in both cancers, and considered TIMM8A as a biomarker to predict the efficacy of this therapy in BRCA and UCEC. The results of TIMM8A enrichment analysis showed us that abnormally expressed TIMM8A might affect the mitochondrial protein in BRCA and UCEC.

**Conclusions:**

Contributed to illustrating the value of TIMM8A as a prognostic biomarker, our findings suggested that TIMM8A was correlated with prognosis and immune infiltration, including CD8+ T cells, Th2 CD4+ T cells, and macrophages in BRCA and UCEC. In addition, TIMM8A might affect immune infiltration and prognosis in BRCA and UCEC by affecting mitophagy. We believed it could also be a biomarker to predict the efficacy of anti-PD-L1 therapy and proposed to improve the efficacy by eliminating MDSC and TAM M2.

**Supplementary Information:**

The online version contains supplementary material available at 10.1186/s12957-022-02736-6.

## Introduction

BRCA is one of the most common malignant tumors in women, accounting for 31% of systemic malignant tumors in women. Since 2014, the prevalence has slowly increased by about 0.5% per year [1]. According to statistics, there are about 2,000,000 new breast cancer patients worldwide each year, and an estimated 620,000 patients die from this cancer [2]. Uterine corpus endometrial cancer (UCEC) is a kind of epithelial malignant tumor that occurs in the endometrium. It is one of the three major malignant tumors of the female reproductive tract, accounting for 7% of female systemic malignant tumors and 20–30% of female reproductive tract malignant tumors. National statistics show that about 142,000 women worldwide develop endometrial cancer each year, and an estimated 42,000 women die from this cancer [3].

The pathogenic process of BRCA and UCEC is related to immunity. It is currently believed that the most important feature that BRCA achieve the purpose of immune evasion is the expression of immunosuppressive receptors (such as cytotoxic T lymphocyte-associated protein (CTLA)-4, programmed cell death protein (PD)-1, lymphocyte activation genes), infiltration of immunosuppressive factors (such as TGF-β and IL-10), and myeloid-derived immunosuppressive cells (MDSC) [4]. In addition, Mamessier found that in BRCA, the function of NK cells was altered. This is also an important mechanism of BRCA to achieve immune evasion [5]. In UCEC, PD-L1 is present in 70–80% of malignant endometrial cells, indicating that the PD-1/PD-L pathway may be an important mechanism for immune evasion in UCEC [6].

Since immune-related mechanisms play an important role in BRCA, immunotherapy strategies are regarded as novel non-surgical treatments for malignant tumors. The clinical success achieved through the use of blocking antibodies against immune checkpoints such as CTLA-4, PD-1, and PD-L1, as well as chimeric antigen receptor (CAR) T cells, represents the potential of immunotherapy in the treatment of cancer [7].

Programmed cell death ligand 1 (PD-L1) is an immune checkpoint that binds to PD-1 expressed on the surface of immune effector cells to produce an immunosuppressive effect [8]. The PD-1/PD-L1 pathway plays a role in maintaining peripheral T lymphocyte tolerance and regulating inflammation. In cancer, the expression of PD-L1 is one of the major immune escape mechanisms. Numerous studies have shown the efficacy of blocking PD-1 or PD-L1 with specific antibodies.

However, the current immunotherapy for BRCA and UCEC is unsatisfactory. BRCA had an overall response rate (ORR) of 24% to the PD-L1 antagonist atezolizumab [9]. Current immunotherapy for UCEC is less effective, with an overall response rate (ORR) of 13% for UCEC to the PD-L1 antagonist avelumab, and a few cases have serious adverse reactions [10]. In addition, many studies have implied that tumor-infiltrating lymphocytes such as tumor-associated macrophages (TAM) and tumor-infiltrating neutrophils (TIN) affect the efficacy and prognosis of non-surgical cancer treatment [11]. However, Nakayama proposed the use of phospho-STAT1 (Phospho-STAT1) as a biomarker to predict the efficacy of anti-PD-L1 therapy in BRCA [12]. In UCEC, research in this area is still relatively blank. If a biomarker can be identified to predict the efficacy of anti-PD-L1 therapy in BRCA and UCEC, it will be of great help to clinical research and drug development

First reported in 2003, TIMM8A is a protein-coding gene located on the X chromosome. Tim8a is a member of the intermembrane space chaperone network [13] and functions in a chaperone-like manner with the chaperone protein Tim13 in the complex, to promote entry of nuclear-encoded precursor proteins into the inner mitochondrial membrane [14]. Studies have shown that pathogenic mutations in TIMM8A result in decreased expression of Tim8a, resulting in defects in nuclear-encoded function and mitochondrial morphology and dysfunction [15]. Studies on mitochondrial morphological changes have proven that mitochondrial morphological changes lead to the reduction of mitophagy, thus accelerating the apoptosis of immune cells [16]. Based on previous findings, we speculated that abnormal expression of TIMM8A in cancer might also affect the function of immune cells by affecting mitochondria, thereby affecting the development and prognosis of cancer. Therefore, in this study, we analyzed the impact of TIMM8A in BRCA and UCEC on multiple immune cells and mitochondria-related pathways and analyzed its potential as a biomarker.

In this study, for the first time, we comprehensively analyzed the expression of TIMM8A and its association with the prognosis of cancer patients using databases such as Ualcan, PrognoScan, Human Protein Atlas, and Kaplan-Meier plotter. In addition, we investigated the association of TIMM8A with tumor-infiltrating immune cells in BRCA and UCEC through the Tumor Immunity Estimation Resource (TIMER) and TIDE databases. The findings in this report elucidate the important role of TIMM8A in BRCA and UCEC, provide potential relationships and underlying mechanisms between TIMM8A and tumor-immune interactions, and provide insights into the use of anti-PD-L1 therapy in BRCA and UCEC. Outcomes in BRCA and UCEC were predicted and recommendations for improvement were made. Finally, we analyzed the functions and pathways of TIMM8A mutation and its 50 frequently altered neighboring genes in BRCA and UCEC patients to investigate the underlying molecular mechanisms of TIMM8A in the pathogenesis or the clinic.

## Materials and methods

### TIMER database analysis

TIMER is a database systematically analyzing different types of tumors (http://timer.comp-genomics.org/) [17], which provides immune infiltrates’ abundances estimated by multiple immune deconvolution methods. We used the Gene plate of Immune Association module to analyze TIMM8A expression in BRCA and UCEC and the correlation of TIMM8A expression with the abundance of immune infiltrates module, including B cells, Th2 CD4+ T cells, CD8+ T cells, macrophages, neutrophils, dendritic cells (DCs), NK cells, and Tregs. In addition, we also used the Cancer Exploration module to obtain the relationship between BRCA and UCEC and protein PINK1 and Parkin.

### PrognoScan database analysis

PrognoScan database is a powerful platform for evaluating potential tumor markers and therapeutic targets (http://www.prognoscan.org) [18]. This web employs the minimum *P*-value approach for grouping patients for survival analysis. We used PrognoScan to search for the relation between gene expression and prognoses of BRCA and UCEC patient including overall survival (OS), distant metastasis survival (DMFS), and disease-free survival (DFS) across a large collection of publicly available cancer microarray datasets. The threshold was adjusted to a Cox *p*-value< 0.05.

### Kaplan-Meier plotter database analysis

The Kaplan Meier plotter (http://kmplot.com/analysis/) [19] is capable to assess the correlation between the expression of 30k genes (mRNA, miRNA, protein) and survival in 25k+ samples from 21 tumor types including breast, ovarian, lung, and gastric cancer. The correlation between TIMM8A expression and survival in BRCA and UCEC was analyzed by Pan-cancer module via mRNA RNA-seq in Kaplan-Meier Plotter.

### UALCAN database analysis

UALCAN is a canceromics data analysis tool (http://ualcan.path.uab.edu/) [20] built on PERL-CGI with high-quality graphics using javascript and CSS. In this study, the TCGA module in UALCAN was used to analyze the expression difference of TIMM8A between normal tissue and BRCA tissue and UCEC tissue, and the expression difference between BRCA and UCEC in different stages. The difference in transcriptional expression was compared by Student’s *t*-test and *P* <0.05 was considered as statically significant.

### TIDE database analysis

TIDE represents tumor immune dysfunction and rejection (http://tide.dfci.harvard.edu/query/) [21], a computational framework developed to evaluate the potential of tumor immune evasion from the gene expression profiles of cancer samples. It has two modules: predict Response and Query Genes. In this study, we use the Query Genes module to obtain the results of TIMM8A immunotherapy, and *P* < 0.05 indicated that the difference was statistically significant.

### LinkedOmics database analysis

LinkedOmics is an open portal (http://www.linkedomics.org/admin.php) [22], including multi-group data of all 32 TCGA cancer types and 10 cancer cohorts of the Clinical Proteomics Cancer Analysis Association (CPTAC). Web applications have three analysis modules: LinkFinder, LinkInterpreterter, and LinkCompar. Its analysis results can be visualized by scatter plot, box plot, or Kaplan-Meier plot. We used the three modules of the website to analyze the high visibility biological process of TIMM8A and obtained the biological process most closely related to TIMM8A.

### The Human Protein Atlas

The Human Protein Atlas (www.proteinatlas.org) [23] is a website about Proteome analysis based on 27,173 antibodies targeting 17,268 unique proteins (proteinatlas.org). We used this website to measure the prognosis of TIMM8A in UCEC pathology and ribonucleic acid and obtained its survival data. The best expression cut-off was selected automatically by the website.

## Results

### The mRNA expression levels of TIMM8A in different types of human cancers

**Fig. 1:**
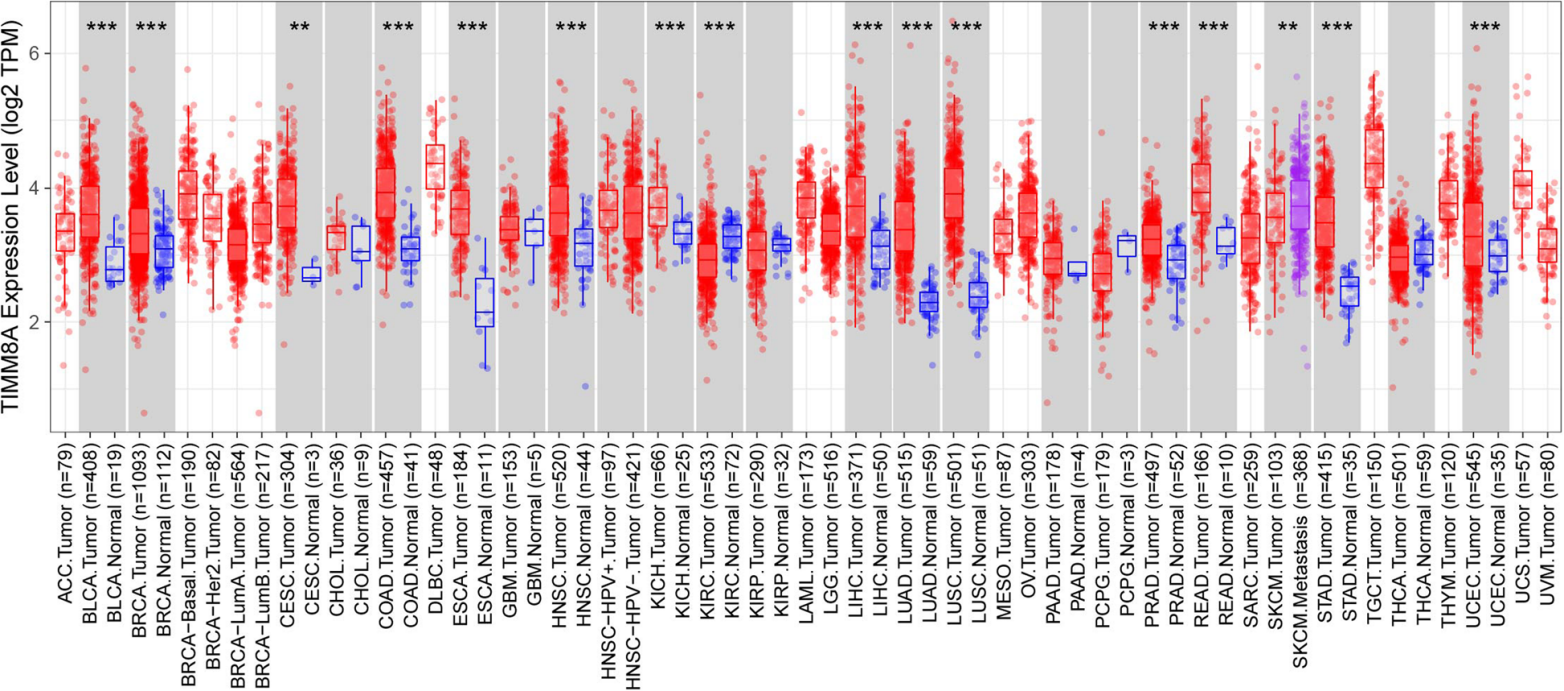
Expression of TIMM8A in different cancers. Human TIMM8A expression levels in different tumor types from TCGA database were determined by TIMER (*P < 0.05, **P < 0.01, ***P < 0.001)

To determine the differences of TIMM8A expression in tumor and normal tissues, the expression of TIMM8A was examined using RNA-seq data of multiple malignant tumors in TCGA. The expression differences of TIMM8A in BLCA (bladder urothelial carcinoma), BRCA, CESC (cervical and endocervical cancer), COAD (colon adenocarcinoma), ESCA (esophageal carcinoma), HNSC (head and neck cancer), KICH (kidney chromophobe), KIRC (kidney renal clear cell carcinoma), LIHC (liver hepatocellular carcinoma), LUAD (lung adenocarcinoma), LUSC (lung squamous cell carcinoma), PRAD (prostate adenocarcinoma), READ (rectum adenocarcinoma), STAD (stomach adenocarcinoma), UCEC, and corresponding normal tissues were statistically significant (Fig. 1). These results indicated that TIMM8A might play a certain role in the occurrence and development of the above cancers, but its specific impact on survival and prognosis still needed further exploration.

### Prognostic potential of TIMM8A in BRCA and UCEC

**Fig. 2:**
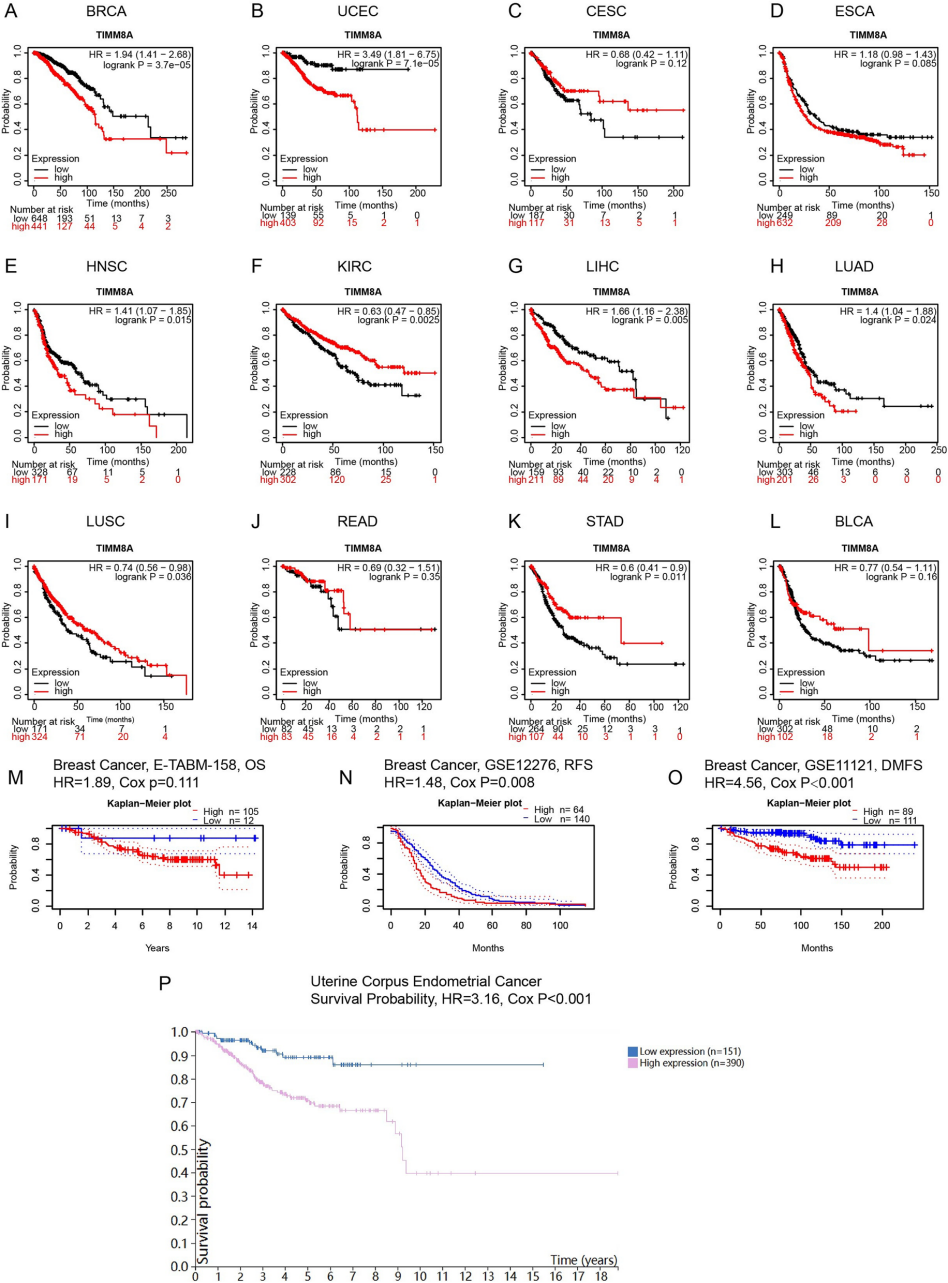
The prognostic potential of TIMM8A in different cancers. **A**–**L** High and low expression of TIMM8A in BRCA, UCEC, CESC, ESCA, HNSC, KIRC, LIHC, LUAD, LUSC, READ, STAD, and BLCA in the Kaplan-Meier plotter database impact on OS. **M**–**O** OS, RFS, and DMFS survival curves in three BRCA cohorts [E-TABM-158 (*n*=117), GSE12276 (*n*=204), and GSE11121 (*n*=200)] in the PrognoScan database. **P** Survival probability in UCEC. OS, overall survival; RFS, recurrence-free survival; DMFS, distant metastasis-free survival (an additional excel file shows this in more detail (see Additional file 1))

To determine the prognostic potential of TIMM8A in the above 15 malignancies, the Kaplan-Meier plotter database was used to evaluate the TIMM8A prognostic value based on Affymetrix microarrays (Fig. 2A–L). Among the above 15 kinds of tumors, the poor prognosis in BRCA (Fig. 2A, OS HR=1.94, *P*=3.7e−05) and UCEC (Fig. 2B, OS HR=1.94, *P*=3.7e−05) was shown to significantly correlate with high TIMM8A expression.

These results inspired us to further explore the effect of TIMM8A on the prognosis of BRCA and UCEC including OS, RFS, and DMFS. Via PrognoScan, we obtained three BRCA cohorts (E-TABM-156 [24, 25], GSE12276 [26], GSE11121 [27–30]) including 118 samples, 204 samples, and 200 samples for survival analysis, the results of which determined that high TIMM8A expression was marginally associated with poor prognosis (Fig. 2M–O, OS HR=1.89, 95%CI=0.86–4.11, Cox *P*=0.111; RFS HR=1.48, 95%CI=1.11–1.98, Cox *P*=0.008; DMFS HR=4.56, 95%CI=2.10–9.91, Cox *P*=0.000127). We investigated the effect of TIMM8A on the survival of UCEC patients using the Human Protein Atlas (Fig. 2P). The results showed that the survival probability of patients in the high expression group was significantly lower than that in the low expression group (HR=3.16, 95%CI=1.721–5.811, Cox *P*<0.001).

### The expression level of TIMM8A in different stages of BRCA and UECE

**Fig. 3:**
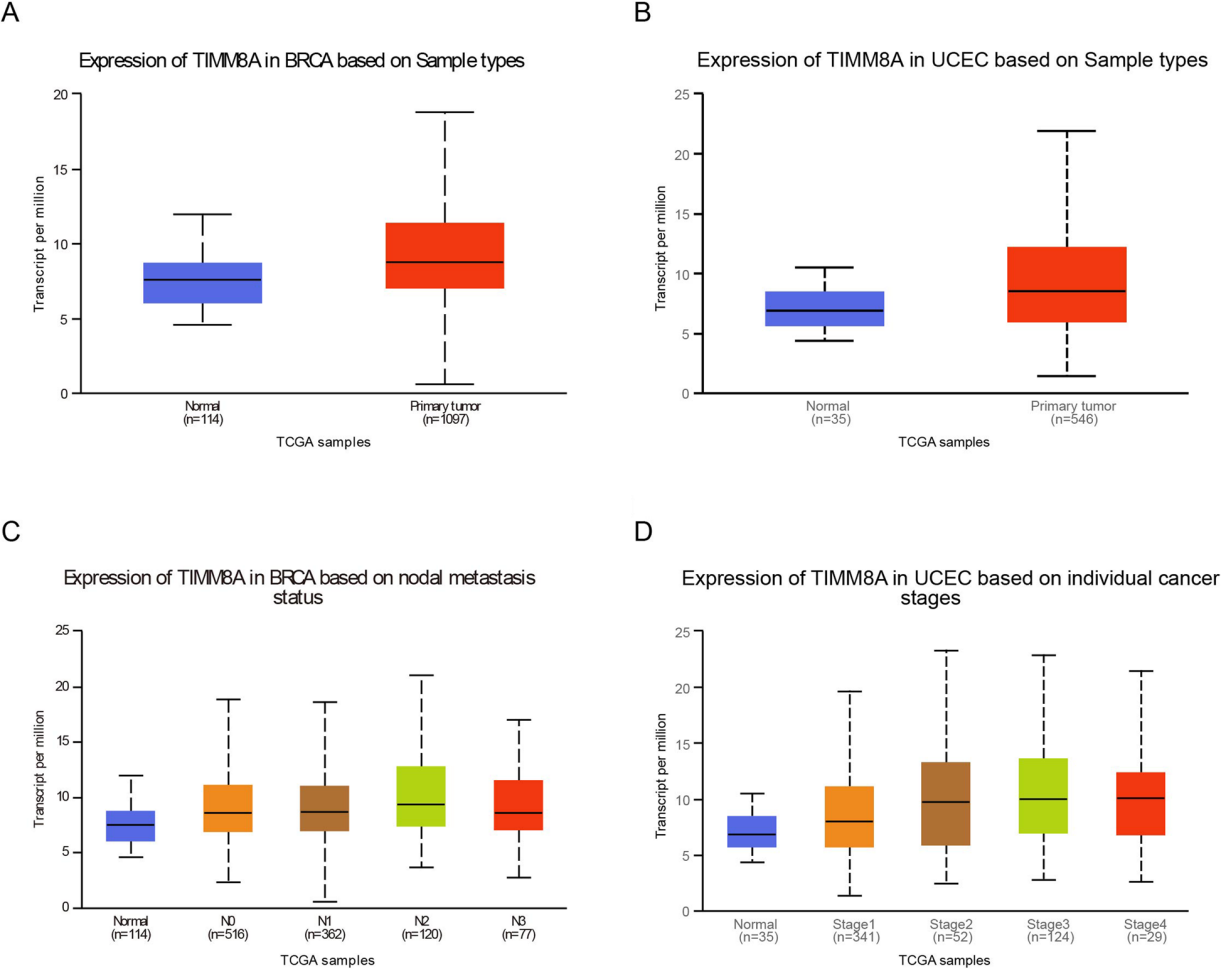
The expression levels of TIMM8A in different stages of BRCA, UCEC, and corresponding normal tissues. **A** Differences of TIMM8A expression levels in BRCA and corresponding normal tissues. **B** Differences of TIMM8A expression levels in UCEC and corresponding normal tissues. **C** Differences of TIMM8A expression levels in different BRCA stage. **D** TIMM8A expression levels of different UCEC stage difference

We then revealed the expression levels of TIMM8A in different stages of BRCA and UCEC and corresponding normal tissues for comparison via the Ualcan database. As shown in Fig. 3A and B, comparing with normal samples, the expression of TIMM8A was upregulated in both BRCA and UCEC (UCEC: normal median=6.876, primary tumor median=8.508; BRCA: normal median=7.552, primary tumor median=8.731). The expression of TIMM8A was increased with an upward trend in all clinical stages of BRCA and UCEC compared with normal tissues (Fig. 3C and D). In BRCA, although the expression of TIMM8A in stage 3 was decreased compared with that in stage 2, it was still higher than that in corresponding normal tissues. The expression level in stage 4 was the highest among all stages (BRCA: stage 1 median = 8.083, stage 2 median = 8.972, stage 3 median = 8.599, stage 4 median = 9.971). In UCEC, the expression level of TIMM8A increased following the cancer stages (UCEC: stage 1 median = 8.089, stage 2 median = 9.817, stage 3 median = 10.024, stage 4 median = 10.095). These results motivated us to explore the relevant mechanisms by which TIMM8A affected the development of the above two cancers.

### Correlation between TIMM8A and PINK1/Parkin pathway protein expression in BRCA and UCEC

**Fig. 4:**
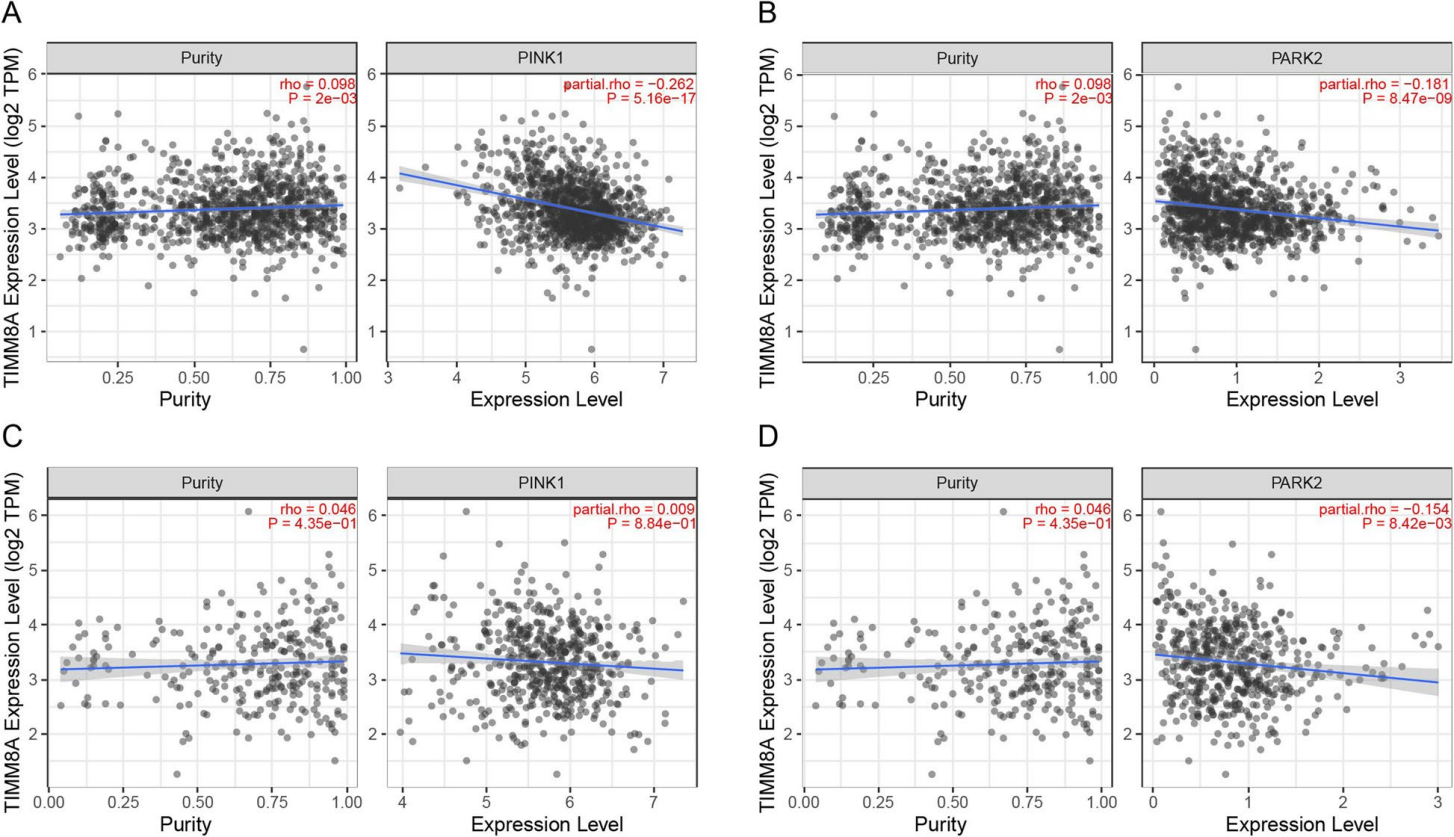
The correlation of TIMM8A with PINK1 and Parkin in BRCA and UCEC. **A** Correlation between TIMM8A and PINK1 in BRCA. **B** Correlation between TIMM8A and Parkin in BRCA. **C** Correlation between TIMM8A and PINK1 in UCEC. **D** Correlation between TIMM8A and Parkin in UCEC

To explore the possible biofunction effect of TIMM8A expression on mitophagy, we used the TIMER database to analyze the correlation of TIMM8A with PINK1 and Parkin in BRCA and UCEC. The results showed that both PINK1 and Parkin were negatively correlated with TIMM8A in BRCA (Fig. 4A and B). In UCEC, Parkin was negatively correlated with TIMM8A with statistical significance (Fig. 4D), whereas there was no significant correlation between PINK1 and TIMM8A (Fig. 4C).

### TIMM8A expression correlated with immune infiltration level in BRCA and UCEC

To explore the mechanism underlying the effect of TIMM8A on BRCA and UCEC survival, we investigated whether TIMM8A expression was associated with immune infiltration levels in these two cancers with the TIMER database. In BRCA, TIMM8A expression level had significant positive correlations with infiltrating levels of B cells (*r* = 0.174, *P* = 3.43e−08), Th2 CD4+ T cells (*r* = 0.564, *P* = 1.89e−84), CD8+ T cells (*r* = 0.147, *P* = 3.02e−06), dendritic cells (*r* = 0.163, *P* = 2.31e−07), macrophages (*r* = 0.254, *P* = 4.83e−16), and neutrophils (*r* = 0.313, *P* = 4.57e−24). In UCEC, the expression level of TIMM8A was positively correlated with Th2-type CD4+ T cells (*r* = 0.329, *P* = 1.78e−03) and had negative correlation with infiltrating levels of CD8+ T cells (*r* = −0.416, *P* = 5.61e−05), macrophages (*r* = −0.338, *P* = 1.30e−03), DC cells (*r* = −0.328, *P* = 1.38e−03), NK cells (*r* = −0.247, *P* = 2.01e−02), and Tregs (*r* = − 0.376, *P* = 3.07e−04). These findings implied that TIMM8A might play a specific role in the immune infiltration of BRCA and UCEC, especially the immune infiltration of Th2-type CD4+ T cells. However, TIMM8A showed a certain positive correlation with the infiltration level of CD8+ T cells in BRCA, but a significant negative correlation in UCEC.

### Analysis of the correlation between TIMM8A and CD8+ T cell infiltration levels in BRCA and UCEC

**Fig. 5:**
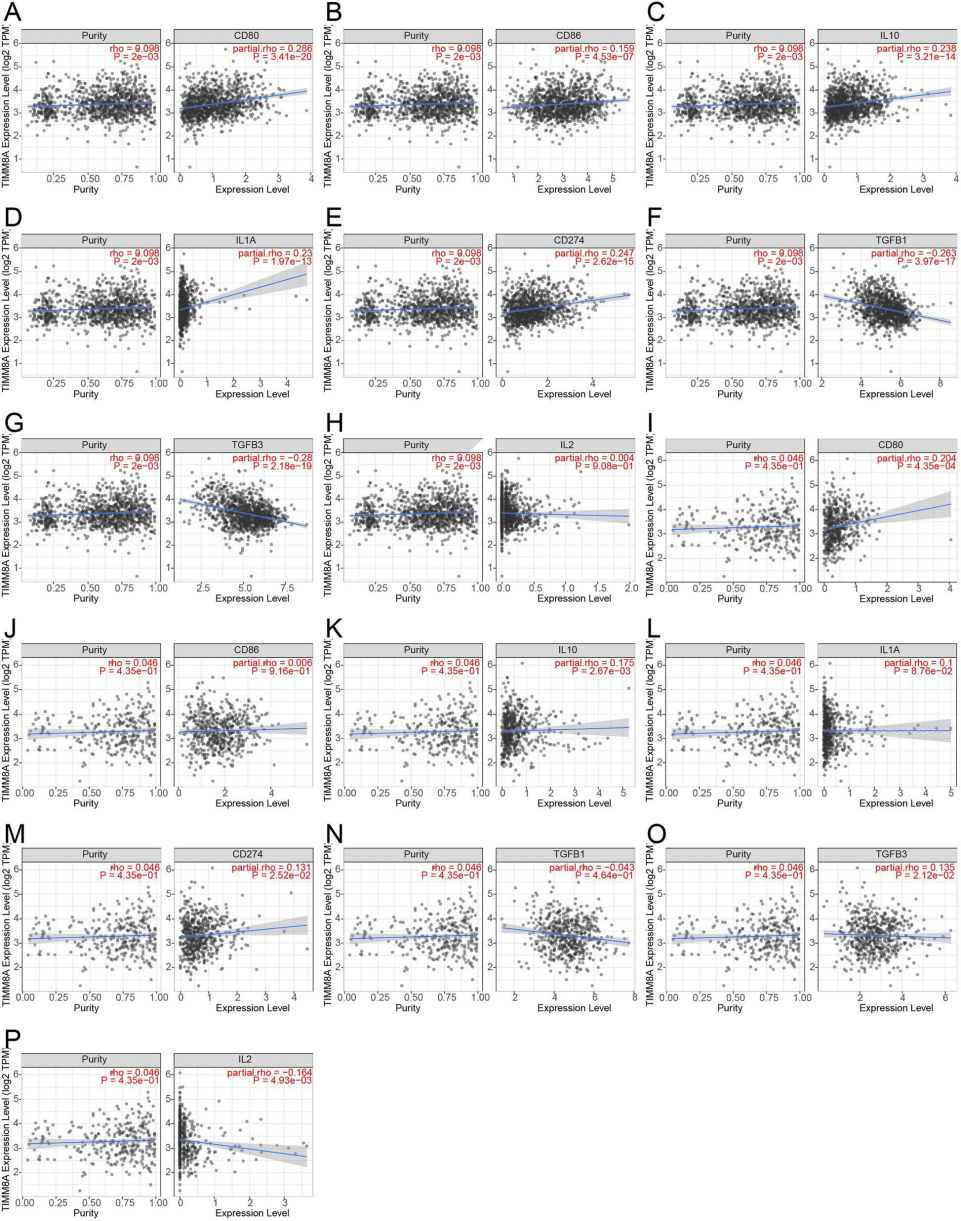
The correlation of TIMM8A expression with various cytokines in BRCA and UCEC. The expression of TIMM8A in BRCA was correlated with the levels of CD80, CD86, IL-10, IL-1A, CD274 (PD-L1), TGFB1, tgfb3, and IL-2 (I-P). The expression of TIMM8a in UCEC was correlated with the levels of CD80, CD86, IL-10, IL-1A, CD274 (PD-L1), TGFB1, TGFB3, and IL-2

As previously described, TIMM8A had opposed associations with CD8+ T cells in BRCA and UCEC, which might be due to the influence of certain cytokines. To this end, we analyzed the association of TIMM8A with certain cytokines in BRCA and UCEC using the TIMER database. The results showed that in BRCA (Fig. 5A–H), the level of TIMM8A was positively correlated with the levels of PD-L1 (CD274), CD80, CD86, IL-10, and IL-1A, while negatively correlated with TFGB1 and TGFB2. In UCEC (Fig. 5I–P), TIMM8A was positively correlated with PD-L1, CD80, IL-10, and TGFB3 and negatively correlated with IL-2 levels.

### Prediction of immune evasion mechanisms in BRCA and UCEC

**Fig. 6:**
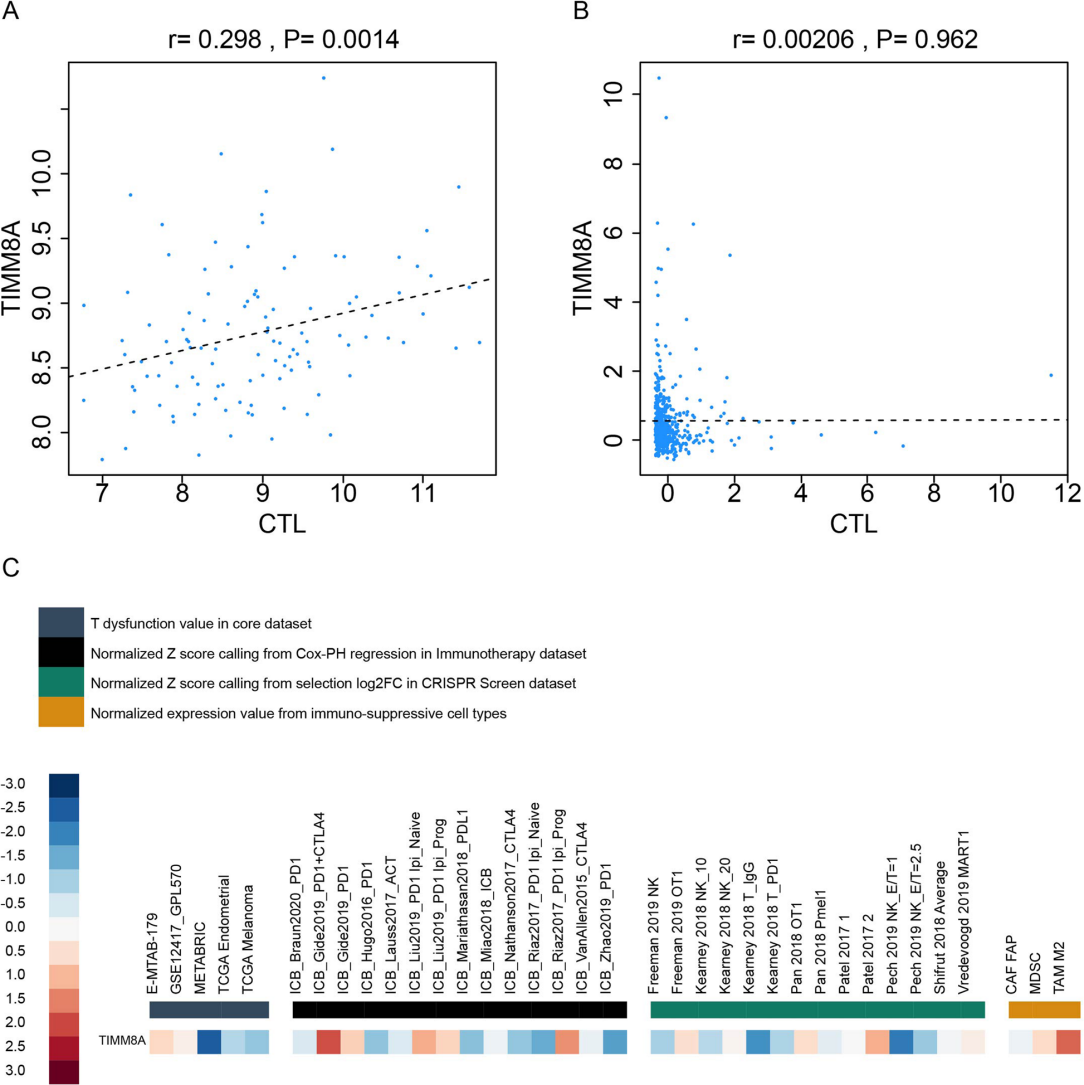
The correlation of BRCA and UCEC with CTL. **A** Relationship between TIMM8A and CTL infiltration in the MetaBric breast cancer dataset. **B** Association of TIMM8A with the degree of CTL invasion in the TCGA endometrial cancer dataset. **C** TIMM8A exhibited a T-cell impairment score of less than zero in the Metabric dataset. It was highly expressed in MDSC and TAM M2 and less expressed in CAF FP. MDSC, myeloid-derived suppressor cells; TAM M2, tumor-associated M2 subtype macrophages; CAF FAP, FAP + tumor-associated fibroblasts

To predict the mechanism of immune evasion in BRCA and UCEC, we explored the correlation of BRCA and UCEC with CTL using the TIDE database. The results showed that TIMM8A was positively correlated with CTL infiltration in BRCA (Fig. 6A), but not in UCEC (Fig. 6B). Figure 6C shows that among the cell types promoting T cell exclusion, myeloid-derived suppressor cells (MDSC) and tumor-associated M2 macrophages (TAM M2) had high expression levels of TIMM8A, whereas CAF FAP had a low level. It showed that in both BRCA and UCEC, TIMM8A might achieve the purpose of immune evasion through T cell exclusion.

### Predicted functions and pathways of the mutations in TIMM8A and their 50 frequently altered neighbor genes in BRCA patients

**Fig. 7:**
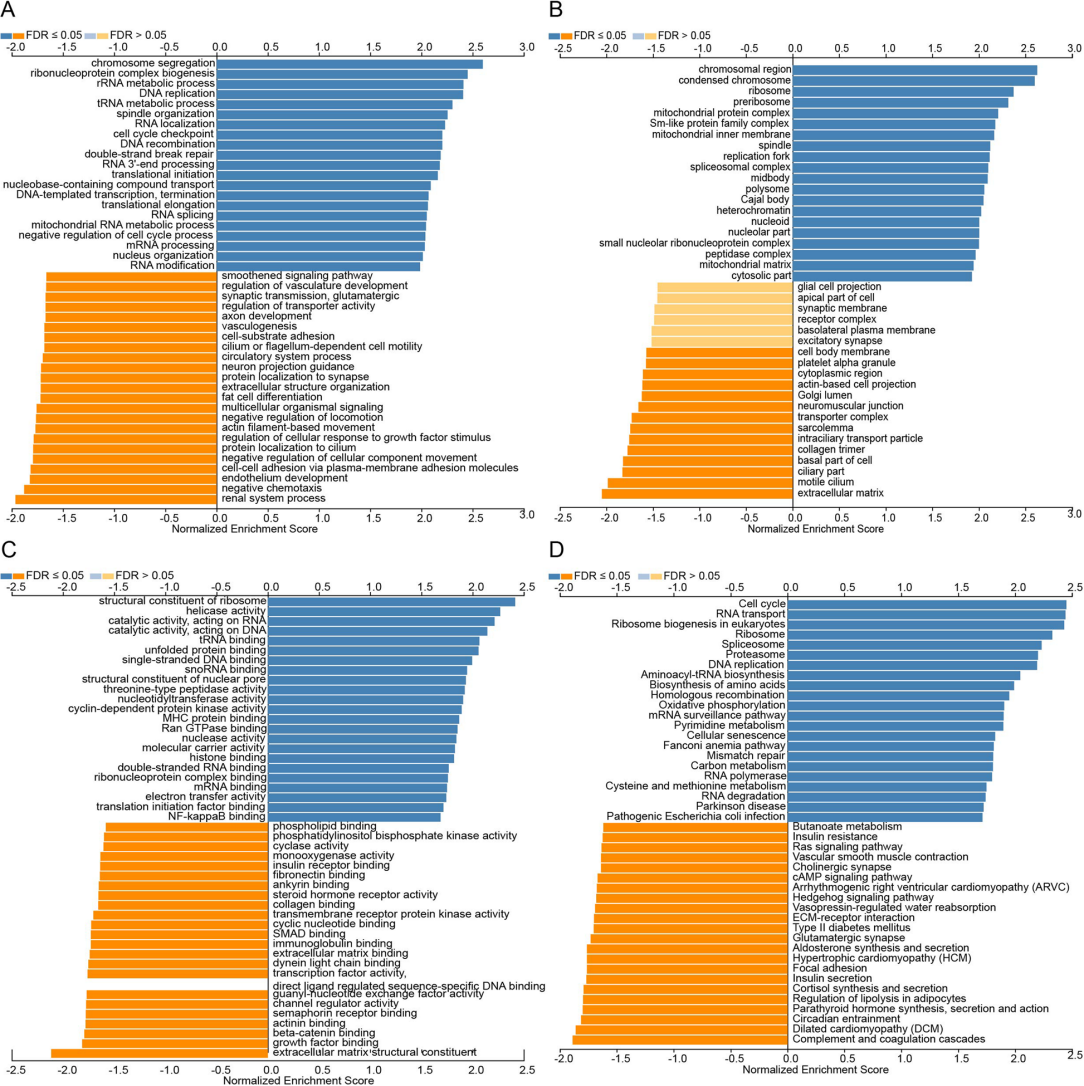
The function of TIMM8A and its 50 frequently changed adjacent genes in BRCA. GO and KEGG functional enrichment analysis predicted three main functions of TIMM8A in BRCA, including biological processes (**A**), cellular components (**B**), molecular functions (**C**), and KEGG pathway analysis (**D**) of TIMM8A in BRCA

We used LinkedOmics website to analyze the function of TIMM8A and its 50 frequently changed adjacent genes. As shown in Fig. 7A, biological processes such as chromosome segregation, ribonucleoprotein complex biogenesis, rRNA metabolic process, DNA replication, and tRNA metabolic process were remarkably regulated by the TIMM8A mutations in BRCA. Cellular components, including chromosomal region, condensed chromosome, ribosome, preribosome, and mitochondrial protein complex, were significantly associated with the TIMM8A alterations (Fig. 7B). In addition, TIMM8A mutations also prominently affected the molecular functions, such as structural constituent of ribosome, helicase activity, catalytic activity, acting on RNA, catalytic activity, acting on DNA, and tRNA binding (Fig. 7C).

In KEGG analysis, 6 pathways including Cell cycle, RNA transport, ribosome biogenesis in eukaryotes, ribosome, spliceosome, and proteasome were associated with the functions of TIMM8A mutations in BRCA (Fig. 7D).

### Predicted functions and pathways of the mutations in TIMM8A and their 50 frequently altered neighbor genes in UCEC patients

**Fig. 8:**
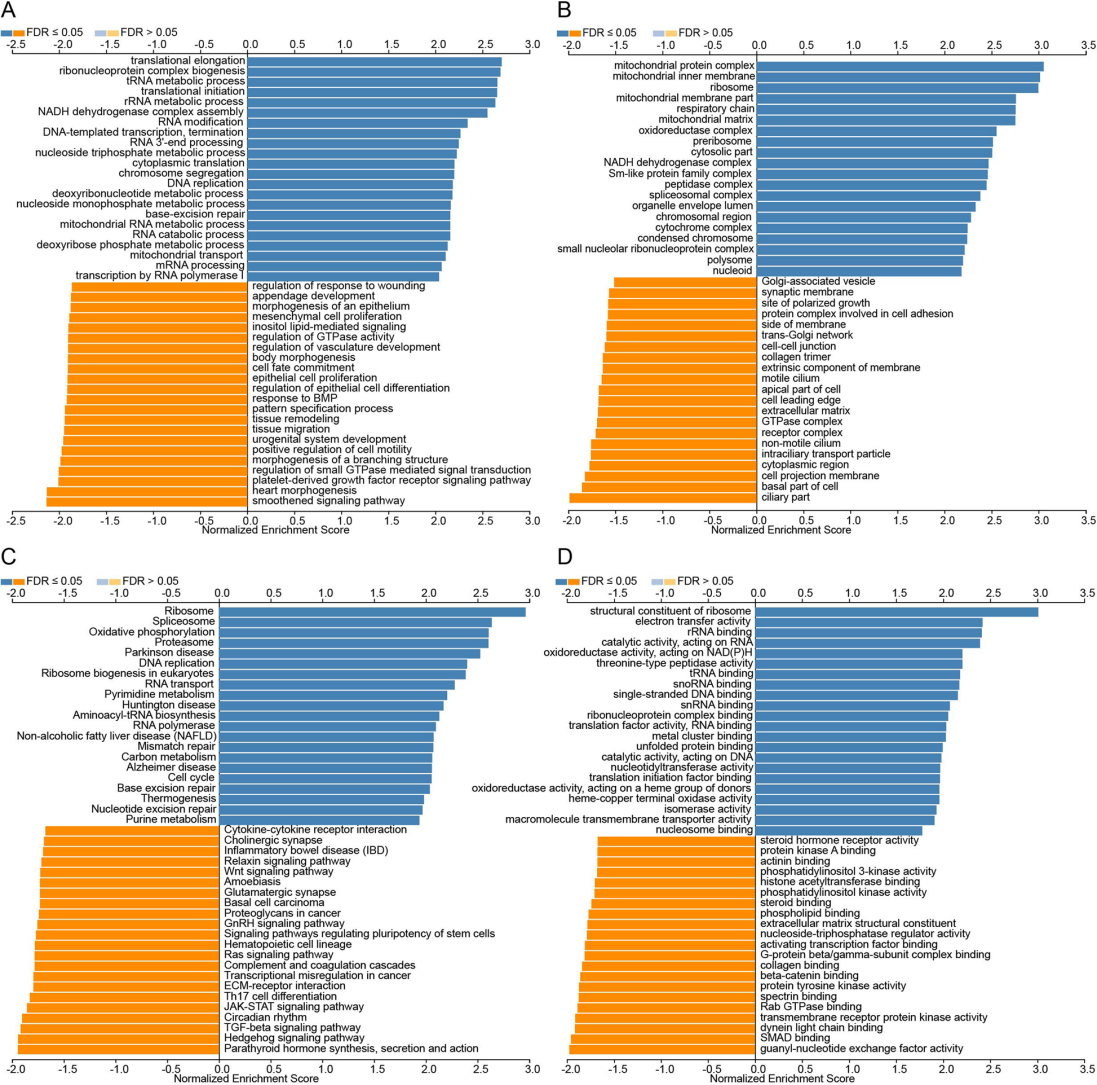
The function of TIMM8A and its 50 frequently changed adjacent genes in UCEC. GO and KEGG functional enrichment analysis predicted three main functions of TIMM8A in UCEC, including biological processes (**A**), cellular components (**B**), molecular functions (**D**), and KEGG pathway analysis (**C**) of TIMM8A in UCEC

We used LinkedOmics website to analyze the function of TIMM8A and its 50 frequently changed adjacent genes. As shown in Fig. 8A, biological processes such as 0ncRNA processing, ribonucleoprotein complex biogenesis, translational elongation, rRNA metabolic process, and translational initiation were remarkably regulated by the TIMM8A mutations in UCEC. Cellular components, including mitochondrial protein complex, ribosome, mitochondrial inner membrane, mitochondrial matrix, and mitochondrial membrane part, were significantly associated with the TIMM8A alterations (Fig. 8B). In addition, TIMM8A mutations also prominently affected the molecular functions, such as structural constituent of ribosome, rRNA binding, electron transfer activity, catalytic activity, acting on RNA, threonine-type peptidase activity (Fig. 8D).

In KEGG analysis, 6 pathways including ribosome, oxidative phosphorylation, spliceosome, proteasome, Parkinson disease, and DNA replication were associated with the functions of TIMM8A mutations in UCEC (Fig. 8C).

## Discussion

BRCA and UCEC are relatively common gynecological cancers that seriously threaten women’s life and health PD-L1 is an immune checkpoint, and its combination with PD-1 is the key to immunosuppression. Many studies have shown the efficacy of blocking PD-1 or PD-L1 using specific antibodies. In hepatocellular carcinoma, the efficacy of the PD-L1 monoclonal antibody Atezolizumab reached 36% [31]. However, in BRCA, immunotherapy by anti-PD-L1 was less than 24% effective [31]. In UCEC, the effective rate is only about 13%, and a few cases have more serious adverse reactions [10].

TIMM8A is a protein-coding gene on the X chromosome that has not been studied in BRCA or UCEC. Changes in its expression can lead to significant changes in mitochondrial morphology affecting mitophagy. Since mitophagy played an important role in decreasing immune cell apoptosis as well as tumorigenesis, we speculated that TIMM8A might affect the prognosis and lymphocyte infiltration levels in BRCA and UCEC. Perhaps it could serve as a biomarker for prognosis and predicting the efficacy of anti PD-L1 which is associated with T lymphocyte infiltration.

In this study, we observed a statistically significant difference in the expression levels of TIMM8A between 15 cancers and corresponding normal tissues using the RNA-seq data of various malignant tumors in the TIMER database. After evaluating the effect of TIMM8A on the survival of 15 cancers using the Kaplan-Meier plotter, we found that the highly expressed TIMM8A had the most significant effect on the survival of BRCA and UCEC, and with the PrognoScan database we reconfirmed that the highly expressed TIMM8A had the most significant adverse effect on the RFS and DMFS of BRCA. The results in the Human Protein Atlas also confirmed the adverse effect of high expression of TIMM8A on the survival rate of UCEC.

Subsequently, we retrieved the expression levels of TIMM8A in different stages of BRCA and UCEC in the Ualcan database. The results showed that in BRCA, except for stage 3, the TIMM8A expression in the other stages was increased as the increase of the stage. While in UCEC, the expression of TIMM8A increased following the cancer stages. This finding suggested that TIMM8A might be used as a prognostic biomarker in BRCA and UCEC.

TIMM8A is closely related to mitochondria which are involved in multiple aspects of tumorigenesis. Xu has found that immune cells such as NK cells can resist apoptosis through mitophagy [16]. Suspecting that TIMM8A might affect immune infiltration by affecting mitophagy, we explored the relationship between TIMM8A and mitophagy. The PINK1/PARKIN pathway plays an important role in mitophagy. We found that in BRCA, the expression of TIMM8A was negatively correlated with PINK1 and Parkin. In UCEC, Parkin was found to be negatively correlated with TIMM8A which was not significantly correlated with PINK1. The PINK1/Parkin pathway can selectively degrade mitochondria and play an important role in the activation of mitophagy [32]. Downregulation of these two proteins can inhibit mitophagy. Since mitophagy is an anti-apoptotic pathway of immune cells, it might affect immune infiltration and prognosis in addition, when mitophagy is inhibited in tumors, the proportion of dysfunctional mitochondria in tumors increases, which reduces mitochondrial oxidative phosphorylation increasing ROS production [33]. Glycolysis was also upgraduated and thus might contribute to the Warburg effect promoting tumorigenesis. This suggested that in both BRCA and UCEC, mitophagy might be inhibited due to the increased expression of TIMM8A, which promoted immune cell apoptosis and tumorigenesis. It might also account for the fact that TIMM8A had a strong negative correlation with NK cell infiltration in UCEC but not in BRCA.

**Fig. 9:**
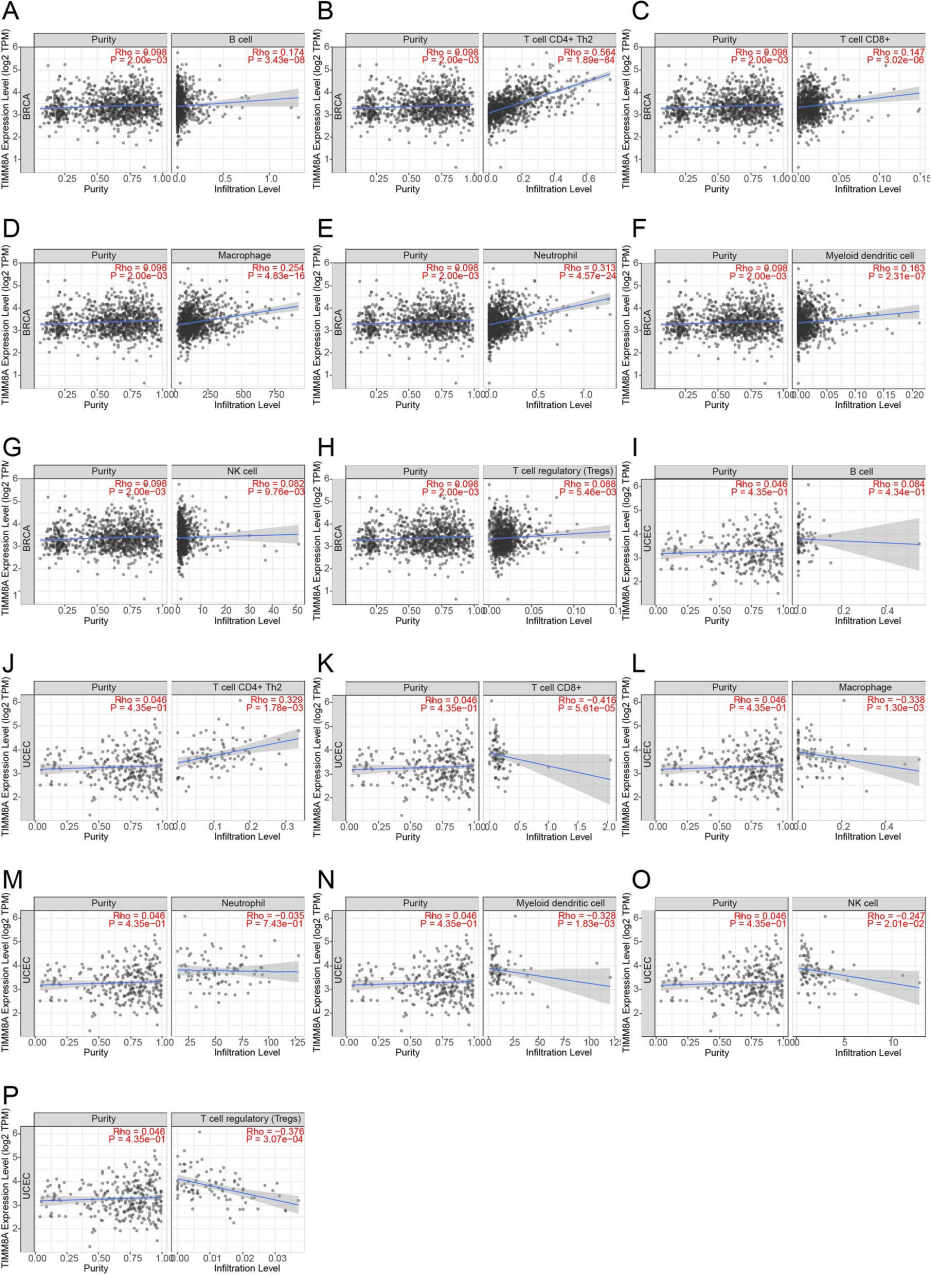
The correlation of TIMM8A expression with immune infiltration levels in BRCA and UCEC. **A**–**H** Relationship between TIMM8A and the degree of infiltration of B cells, Th2 CD4+ T cells, CD8+ T cells, macrophages, neutrophils, DC cells, NK cells, and Tregs cells in BRCA. **I**–**P** The relationship between TIMM8A and the infiltration of B cells, Th2-type CD4+ T cells, CD8+ T cells, macrophages, neutrophils, DC cells, NK cells, and Tregs cells in UCEC

The correlation between TIMM8A and various immune cell infiltration in BRCA and UCEC was verified by Fig. 9, especially the high positive correlation between Th2 CD4+ T cells and TIMM8A. Studies have proved that NK cells can prevent tumor growth directly by killing tumor cells or indirectly by producing mediators with anti-angiogenic properties [34]. CD8+ T cells play an extremely important specific immune role in the process of fighting tumors [35]. However, MDSCs can induce T lymphocyte dysfunction through direct cell-cell contact and the production of immunosuppressive mediators [36–38]. Th2 CD4+ T cells and B cells also limit antitumor immunity by inhibiting Th1 and CTL responses while promoting humoral immunity [39]. In BRCA and hepatocellular carcinoma, Zhang et al. [40] and Zhu et al. [41] also have certified that Th2 CD4+ cells are associated with poor prognosis. We speculated that Th2 CD4+ T cells were a common key factor in the poor prognosis of the BRCA and UCEC. In UCEC, TIMM8A showed a strong negative correlation with both CD8+ T cells and NK cells, which might be an important reason for the higher HR value of TIMM8A in UCEC than in BRCA.

Among the cytokines tested in Fig. 5, CD247 (PD-L1), CD80, CD86, IL1-A, and IL-2 promoted the proliferation of CD8+ T cells. As anti-inflammatory factors, IL-10 and TGF-β can inhibit the activation and proliferation of CD8+ T cells. Th2 CD4+ T cells can indirectly suppress cellular immunity by inhibiting the proliferation of Th1 CD4+ T cells. The results in Fig. 9 showed that TIMM8A was significantly positively associated with Th2 CD4+ T cells in both cancers, which might also affect the level of CD8+ T cell infiltration. CD80 and CD86 are costimulatory signaling molecules for T cells, which can help T cells activate. IL-1 and IL-2 are essential for the proliferation of activated CD8+ T cells. In BRCA, the TIMM8A level was positively correlated with IL-1A which could help increase the number of CD8+ T cells. However, in UCEC, the level of TIMM8A was negatively correlated with IL-2. In addition, the positive correlation of CD80 and CD86 with TIMM8A in BRCA was stronger than that in UCEC. Not only that, in the two cancers, the most significant correlation difference was the TGF-β gene (TGFB1, TGFB3). In BRCA, TIMM8A showed a strong negative correlation with TGFB1 and TGFB3. In UCEC, however, TIMM8A was positively correlated with TGFB3. This might be the main reason why the expression of TIMM8A in BRCA had a certain positive correlation with CD8+ T cells, but it had a significant negative correlation in UCEC.

PD-L1 has an inhibitory effect on CD8+ T cell activation to CTL, as well as CTL’s toxicity and proliferation. However, the positive correlation between TIMM8A and PD-L1 in UCEC was weaker than that in BRCA. This might lead to differences in the degree of CTL infiltration associated with TIMM8A in the two cancers and might be related to the mechanisms of immune evasion of BRCA and UCEC. To test this conjecture, we needed to predict the mechanism by which BRCA and UCEC performed immune evasion.

As mentioned above, the infiltration of CD8+T and CTL cells both showed a certain positive correlation with TIMM8A in BRCA. For satisfactory anti-PD-L1 therapy efficacy, an important condition is high CTL infiltration in the tumor [42]. Since TIMM8A was highly expressed in BRCA, it might be a good indicator of immune response to anti-PD-L1 therapy in BRCA. The poor efficacy of current treatment in BRCA may be affected by MDSC and TAM M2. Previous studies reported that MDSC limit the efficacy of anti-PD-L1 therapy which was improved after TAM M2 is eliminated in non-small cell lung cancer [43, 44]. If MDSC and TAM M2 can be eliminated while treating BRCA, the efficacy may be greatly enhanced. However, in UCEC, the expression of TIMM8A had a significantly negative correlation with the infiltration level of CD8+ T cells and was not significantly correlated with CTL infiltration level, so anti-PD-L1 therapy might not achieve good efficacy in UCEC.

Finally, we analyzed the function and pathway of TIMM8A and its 50 adjacent genes in BRCA and UCEC patients and found that surface cell cycle and RNA transport were significantly associated with TIMM8A mutations. The TIMM8A mutation in BRCA significantly regulates molecular functions such as 0ncRNA processing, cell components such as mitochondrial protein complexes, ribosomal structure and composition, and ribosomal pathway. Meanwhile, our results showed that protein translation was significantly related to TIMM8A mutation. TIMM8A mutation in UCEC has significant regulation on chromosome separation, cell components such as chromosome region, molecular function such as ribosomal structure composition, cell cycle, and other pathways. In general, TIMM8A is closely related to mitochondrial composition and function in BRCA and UCEC. It is speculated that TIMM8A may affect the process and prognosis of BRCA and UCEC by affecting mitochondrial function. Previous research has established that the proportion of TIMM8A mutation in the population with auditory neuropathy was 1.8 %, and the deafness-myotension disorder-optic neuropathy (DDON) syndrome was caused by the unassembled DDP1/TIMM8a-TIMM13 complex in mitochondria [45]. In addition, in DDON, there are about 15 pathogenic variants in TIMM8A, which will lead to the replacement of methionine required to start protein translation, resulting in the complete deletion of Tim8a/DDP protein and affecting the function of neurons in specific neuronal groups. This is consistent with the enrichment analysis that TIMM8A has a certain effect on mitochondrial protein complexes. Most importantly, the C-terminal portion of Tim8a interacts with the dynamic-related protein 1 (DRP1) during mitochondrial programmed cell death (PCD), which is characterized by extensive mitochondrial fragmentation during PCD. This is achieved by DRP1 interacting with Tim8 a to locate the mitochondria and increase their fission rate [13]. It indicates that TIMM8A can increase the mitochondrial fission rate. Zhang found that the expression of DRP1 protein was generally increased in BRCA, and the increased amount was directly proportional to the invasiveness and metastasis of BRCA [46]. And mitochondrial fission was enhanced in metastatic BRCA. Cormio et al. found that in UCEC, increased mitochondrial biosynthesis required for mitochondrial fission was considered a potential biomarker for determining the risk of malignant transformation [47]. Zheng et al. found that mitochondrial fragmentation reduced the number of NK cell recapitulation and two major subpopulations of NK cells in the tumor microenvironment, and weakened their antitumor ability [48]. Tim8a can enhance the efficiency of mitochondrial fission and the degree of mitochondrial fragmentation by binding to DRP1. This indicated that TIMM8A might have the same pathogenic pathway in DDON, auditory neuropathy, BRCA, and UCEC. That was, they all affected the disease by affecting mitochondrial function and fission.

Previous studies of TIMM8A have been limited to neurodegeneration and deafness-dystonia-optic neuron disease syndromes, with a lack of articles in oncology. This study illustrated that TIMM8A was abnormally expressed in a variety of malignant tumors, and significantly affected the prognosis of BRCA and UCEC for the first time. We further explored the effect of TIMM8A on the level of immune infiltration in BRCA and UCEC and preliminarily speculated that TIMM8A affected immune infiltration in BRCA and UCEC by affecting mitophagy, thereby leading to the poor prognosis of these two cancers. In addition, this study predicted the efficacy of anti-PD-L1 therapy against BRCA and UCEC and made recommendations to improve efficacy by eliminating MDSCs and TAM M2. There exists scope for improvement in this study. The effect of TIMM8A on DMFS and RFS of UCEC still needs further exploration. The relationship with other immune-related cytokines is also not fully explored. In future research, we plan to overexpress or knock-down TIMM8A in T cells, NK cells, MDSC, and TAM M2, then evaluate the changes in immune function of these cells with in vivo and in vitro experiments, in order to study the impact of TIMM8A on the progression and treatment of BRCA and UCEC more precisely and accurately.

## Conclusions

Taken together, for the first time, our pan-cancer analyses of TIMM8A indicated statistical correlations of TIMM8A expression with clinical prognosis in BRCA and UCEC. And found that the expression of TIMM8A in BRCA and UCEC substantially increased following the cancer stages, which contributed to illustrate the value of TIMM8A as a prognostic biomarker in BRCA and UCEC. Furthermore, we speculated on the possible mechanism by which TIMM8A affected immune infiltration and prognosis in BRCA and UCEC by affecting mitophagy, providing inspiration for futural drug development. In addition, we predicted the efficacy of anti-PD-L1 therapy in BRCA and UCEC. We believed that TIMM8A could serve as a biomarker predicting the efficacy of anti-PD-L1 therapy. TIMM8A predicts good efficacy in BRCA but not in UCEC. We proposed to improve the efficacy by eliminating MDSC and TAM M2, which would contribute to the development of clinical research in the future.

## Supplementary Information

**Additional file 1.** Raw measurements of survival time of UCEC patients and their TIMM8A expression levels. The raw data showed that the prognosis of the high expression group (FPKM value higher than 2.15) was poorer than the low expression group (FPKM value lower than 2.15).

## Data Availability

The datasets analyzed during the current study are available in the TIMER (http://timer.comp-genomics.org/), UALCAN (http://ualcan.path.uab.edu/), Prognoscan (http://www.abren.net/PrognoScan/), TIDE (http://tide.dfci.harvard.edu/query/), Kaplan-Meier plotter (http://kmplot.com/analysis/), LinkedOmics (http://www.linkedomics.org/admin.php), and the Human Protein Atlas (www.proteinatlas.org). The datasets analyzed during the current study are available from the corresponding author on reasonable request. All relevant data are within the article and supporting material.

## Abbreviations

TIMER: Tumor Immune Estimation Resource
HPA: Human Protein Atlas
TIDE: Tumor Immune Dysfunction and Exclusion
BRCA: Breast cancer
UCEC: Uterine corpus endometrial cancer
MDSC: Myeloid-derived suppressor cells
TAM M2: Tumor-associated M2 macrophages
CTLA-4: Cytotoxic T lymphocyte-associated protein
PD-1: Programmed cell death protein
MDSC: Myeloid-derived immunosuppressive cells
CAR: Chimeric antigen receptor
PD-L1: Programmed cell death ligand 1
ORR: Overall response rate
TAM: Tumor-associated macrophages
TIN: Tumor-infiltrating neutrophils
DCs: Dendritic cells
OS: Overall survival
DMFS: Distant metastasis survival
DFS: Disease-free survival
CPTAC: Clinical Proteomics Cancer Analysis Association
BLCA: Bladder urothelial carcinoma
CESC: Cervical and endocervical cancer
COAD: Colon adenocarcinoma
ESCA: Esophageal carcinoma
HNSC: Head and neck cancer
KICH: Kidney chromophobe
KIRC: Kidney renal clear cell carcinoma
LIHC: Liver hepatocellular carcinoma
LUAD: Lung adenocarcinoma
LUSC: Lung squamous cell carcinoma
PRAD: Prostate adenocarcinoma
READ: Rectum adenocarcinoma
STAD: Stomach adenocarcinoma
CAF FAP: FAP+ cancer-associated fibroblast
ROS: Reactive oxygen species
DDON: Deafness-myotension disorder-optic neuropathy
DRP1: Dynamic-related protein 1
PCD: Programmed cell death
